# Bone physiological adaptations to whole-body vibration in mouse models: A Systematic Review

**DOI:** 10.1371/journal.pone.0353776

**Published:** 2026-07-14

**Authors:** Roberto Bonanni, Ida Cariati, Lucio Caprioli, Cristian Romagnoli, Angela Falvino, Saeid Edriss, Pierangelo Cifelli, Giovanna D’Arcangelo, Elvira Padua, Virginia Tancredi, Giuseppe Annino

**Affiliations:** 1 Department of Human Science and Promotion of Quality of Life, San Raffaele Open University, Rome, Italy; 2 Department of Systems Medicine, “Tor Vergata” University of Rome, Rome, Italy; 3 Sports Engineering Laboratory, Department of Industrial Engineering, “Tor Vergata” University of Rome, Rome, Italy; 4 Centre of Space Bio-Medicine, “Tor Vergata” University of Rome, Rome, Italy; Università degli Studi di Milano: Universita degli Studi di Milano, ITALY

## Abstract

Whole-body vibration (WBV) is known to mechanically stimulate bone tissue, modulating its turnover and microarchitecture. Our systematic review aimed to collect the available evidence on the effects of WBV in mouse models, comparing the different experimental protocols and results obtained. A systematic literature search was conducted in the MEDLINE, Scopus, and Web of Science databases, in accordance with PRISMA guidelines. Experimental studies investigating the effects of WBV on healthy mice of different age groups, models of osteoporosis or fracture healing, and other models of bone loss were included. Methodological quality was assessed using SYRCLE’s RoB tool to analyze the reliability of the results and the risk of bias and the adapted GRADE approach to determine the overall level of certainty of the evidence. Twenty-nine studies were included, of which 10 were on healthy mice, 9 on osteoporotic or fracture healing models, and 10 on other models of bone loss. Overall, WBV improved bone formation, mineral density, and trabecular and cortical microarchitecture, although the results were heterogeneous and dependent on frequency, acceleration, duration, and recovery times. In some models, WBV combined with parathyroid hormone (PTH) or oestrogen showed synergistic effects, while in disuse and metabolic models, mechanical vibrations preserved bone mass and reduced osteoclastic activity. WBV represents a potentially useful strategy for modulating bone turnover and improving density and microarchitecture, with effects strongly influenced by protocol parameters and pathophysiological status. Further studies are needed to clarify the mechanisms, optimize protocols, and evaluate the clinical impact in different study populations.

## Introduction

Numerous factors, such as lifestyle, pathological conditions, and pharmacological interventions, can dramatically compromise bone tissue integrity, promoting the onset of fractures and contributing to disability and mortality [1]. In such circumstances, preserving bone health could increase both life expectancy and quality of life, significantly reducing the socio-economic burden associated with the management of fragility fractures [2,3]. Although established strategies, such as anti-osteoporotic drugs and physical exercise, can help slow bone loss, additional interventions aimed at positively modulating bone metabolism could represent valuable tools for preventing skeletal fragility in various clinical contexts [4,5]. In this regard, numerous authors have investigated the effects of whole-body vibration (WBV) on bone tissue in various study populations [6], often reporting encouraging results [7–11]. Among these, the randomized controlled trial (RCT) by ElDeeb et al. found an improvement in vertebral and femoral bone mineral density (BMD) in postmenopausal women undergoing WBV, suggesting its effectiveness in counteracting osteoporotic bone loss [12]. In addition, the systematic review and meta-analysis by Oliveira and colleagues revealed heterogeneous effects on BMD in postmenopausal women, with improvements primarily in the lumbar spine and femur observed only under specific intervention conditions and in subgroup analyses [13]. Similarly, Massini et al. conducted a systematic review and meta-analysis of RCTs involving 202 older adults, observing a significant effect of WBV on total femoral BMD, while no significant changes were detected in the femoral neck or lumbar spine [10]. Furthermore, the systematic review and meta-analysis by Jepsen and colleagues, which included 14 RCTs involving adults ≥50 years of age, showed a reduction in the fall rate, while the effects on BMD and bone microarchitecture were generally inconclusive [14]. On the other hand, Baker and colleagues evaluated the effect of WBV in reducing bone resorption in breast cancer patients undergoing aromatase inhibitor therapy, finding no significant differences between the groups [15]. Therefore, the effectiveness of WBV in maintaining bone mass requires further investigation, as the currently available evidence remains inconclusive. In fact, a first critical issue associated with WBV is the lack of universally recognized protocols for specific populations. In this regard, exposure time and recovery time between two consecutive sessions could influence the benefits of WBV, in addition to the parameters that characterize vibrating platforms, such as frequency, acceleration, and shift. Secondly, as bone loss is multifactorial, the effect of WBV in various conditions should be considered to inform whether it should be promoted or advised against in specific circumstances. In this context, the integration of preclinical evidence takes on particular importance, as a thorough analysis of research findings from mouse models of bone loss can provide concrete help both in selecting the most effective protocols and tools and in assessing the conditions of bone loss in which WBV may or may not be of significant benefit. In fact, mouse models represent a highly significant experimental approach, allowing for the investigation of site-specific, cellular, and molecular responses that cannot be directly studied in humans [16,17]. Furthermore, mice represent a widely used experimental system in bone and WBV research compared to other experimental models, constituting a well-established approach that enhances comparability across studies and facilitates the interpretation of the mechanisms underlying skeletal adaptations to mechanical stimuli.

However, despite the growing number of preclinical studies, the current literature still lacks a comprehensive synthesis of WBV-induced bone adaptations in mouse models, thus representing a significant gap in this field. Consequently, to the best of our knowledge, this is the first systematic review with the primary objective of summarizing the effects of WBV on bone outcomes in mouse models, including both healthy mice and models of bone loss. Second, our systematic review aims to describe the different protocols used and the experimental results obtained to identify the characteristics of the most effective WBV training programmes for maintaining and improving bone health. This type of analysis could aid future research in both the preclinical field, by suggesting appropriate vibratory training protocols, and in clinical trials evaluating the adoption of WBV as an additional strategy for reducing bone loss in specific circumstances.

## Methods

### Eligibility criteria, source information, and search strategy

Our systematic review, which summarizes the evidence on the effects of WBV on bone tissue in various mouse models, was conducted in strict accordance with the Preferred Reporting Items for Systematic Reviews and Meta-Analyses (PRISMA) guidelines (S1 Table), to ensure maximum transparency and reproducibility of the methodological approach [18]. The study protocol, which was drawn up prior to the systematic literature review, has been registered in the open science framework (OSF) repository (DOI: 10.17605/OSF.IO/VS8DP) to ensure methodological transparency and strengthen the integrity of the review process.

Particularly, the systematic literature search was conducted on 2 January 2026 by consulting the three electronic databases MEDLINE, Scopus, and Web of Science (S2 Table). The search strategy adopted was based on the following combination of keywords: ((Whole Body Vibration) OR (Whole-Body Vibration) OR (WBV) OR (Vibratory Training)) AND ((Musculoskeletal system) OR (Bone) OR (Bone Mineral Density) OR (BMD) OR (Bone Loss) OR (Bone Mineral Content)) AND ((Mouse Models) OR (Murine Models) OR (Mice)). This formulation was designed to identify terms relevant to mechanical vibration, bone parameters, and mouse models. No restrictions were imposed regarding language or open access, thereby increasing the number of studies included, ensuring a more comprehensive coverage of the available literature, and reducing the potential risk of bias arising from language barriers or resource constraints. The final selection included only experimental studies, while narrative reviews and conference papers were excluded. The eligibility of studies was determined using the PICOS (Population, Intervention, Comparison, Outcome, and Study design) approach, applying the inclusion and exclusion criteria outlined in Table 1.

**Table 1:** PICOS criteria for inclusion and exclusion of studies.

CATEGORY	INCLUSION CRITERIA	EXCLUSION CRITERIA
**Population**	Mouse models, including healthy mice or models of bone loss, without restrictions on age, sex, or strain	Species other than mice (rats, other rodents)
**Intervention**	Exposure to WBV, alone or in combination with other interventions, delivered using any device or parameters (session duration, total duration, recovery period, frequency, acceleration, shift)	- Interventions not involving WBV- Localized vibrations not qualifying as WBV
**Comparison**	- Control group (sedentary or exposed on an inactive platform) - Group exposed to other intervention	- Studies without a valid comparator group- Comparisons that do not allow meaningful interpretation
**Outcome**	Measures of bone mass and microarchitecture, biochemical markers of bone metabolism, biomechanical properties, and histomorphometry	- Studies without bone-related outcomes - Outcomes not relevant to bone health
**Study design**	Experimental in vivo studies conducted on mice	All other study designs

PICOS: population, intervention, comparison, outcome, and study design; WBV: whole-body vibration.

### Selection process, collection and synthesis of data

Two experienced researchers independently conducted the initial bibliographic search using predefined keywords. In accordance with previous studies, all articles retrieved from the three selected databases were uploaded into the Rayyan software and screened for duplicates [19]. Subsequently, titles and abstracts were reviewed separately by the two researchers to exclude studies unrelated to the topic of our systematic review. The eligibility of the full-text articles was then assessed independently by the same two reviewers, based on the previously defined PICOS inclusion and exclusion criteria. Any discrepancies in article selection were resolved through consultation with a third researcher.

For each study included in our systematic review, data on the population, interventions, comparators, outcomes, and experimental design were extracted independently by the two researchers and then compared to verify their completeness and accuracy. Regarding the characteristics of the vibratory stimulus, the main parameters considered a priori as relevant for interpreting effects on bone tissue included vibration frequency, acceleration, amplitude or shift, vibration direction, characteristics of the vibrating platform, session frequency, duration and number of sessions, and recovery intervals between sessions.

The included studies were subsequently classified into three main categories: (i) healthy young, adult and elderly mouse models, (ii) osteoporotic and fracture healing models, (iii) other models of bone loss including disuse, genetic, metabolic, inflammatory and neuromuscular models. Due to the high heterogeneity among the included studies, it was not possible to conduct a meta-analysis or a stratified quantitative synthesis of the results. This heterogeneity was driven by differences in animal models, bone outcomes and, above all, WBV protocols, with variability in the parameters of frequency, acceleration and duration, as well as in the vibrating platform used. In this context, it was not possible to define a single primary outcome, as the included studies reported heterogeneous endpoints that were not directly comparable with one another, including densitometric, microstructural and biomechanical parameters, as well as measures of the expression of markers of bone formation and resorption.

### Quality control and bias risk assessment

The risk of bias and methodological quality of the experimental studies included in the systematic review were independently assessed by three expert reviewers using the RoB tool for animal intervention studies (SYRCLE’s RoB tool). This methodology is an adaptation of the Cochrane RoB tool [20], developed specifically for animal intervention studies and capable of accounting for methodological differences between experimental models and RCTs. Particularly, the SYRCLE’s RoB tool assesses ten domains of potential bias: selection bias (random sequence generation, baseline characteristics, and allocation concealment), performance bias (random housing and blinding), detection bias (random outcome assessment and blinding of outcome assessment), attrition bias (incomplete outcome data), reporting bias (selective reporting) and other bias [21]. Each domain was classified as “low”, “high,” or “unclear” risk of bias based on the information reported in the studies. A “low risk” rating was assigned where there were sufficient methodological details and clearly described procedures; “high risk” where there were methodological issues likely to introduce bias; and “unclear” where the available information was either missing or insufficient to allow for an overall assessment. Any discrepancies between researchers were discussed until a shared decision was reached. In the event of disagreement, a fourth researcher made the final decision.

### Certainty of evidence assessment

A Grading of Recommendations, Assessment, Development, and Evaluation (GRADE) approach adapted for preclinical studies was used by three expert reviewers to independently assess the overall certainty of the evidence. This methodology has been proposed in recent years to improve the assessment of the translational potential and certainty of evidence derived from animal model studies [22]. Specifically, five domains were considered, such as risk of bias, inconsistency, indirectness, imprecision and publication bias. For each outcome, the certainty of the evidence can be classified as high, moderate, low or very low. Due to the high heterogeneity between studies, the overall certainty of the evidence was assessed only for outcomes reported in at least four studies, resulting in an overall certainty of low or very low, in line with what is commonly observed in preclinical studies (S3 Table). Any differences of opinion among the researchers were discussed until a consensus was reached. In the event of a disagreement, a fourth reviewer was consulted to make the final decision.

## Results

### Search results

**Fig 1:**
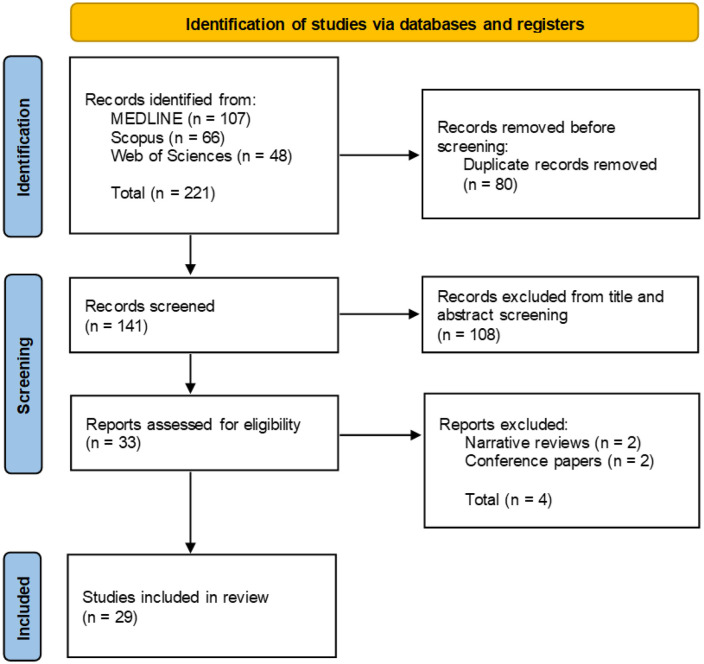
The study selection process is shown in the PRISMA flowchart.

The initial bibliographic search identified a total of 221 articles, including 107 from MEDLINE, 66 from Scopus, and 48 from Web of Science. All records were imported into Rayyan bibliographic reference management software, and 80 articles were excluded as duplicates. Screening of the titles and abstracts of the remaining 141 articles led to the exclusion of 108 studies, as they were not relevant to the objectives of our systematic review. Particularly, several studies were excluded because they were conducted on experimental models other than mice. Some studies were excluded because the applied vibration mode did not correspond to WBV or did not assess the effects of WBV on bone tissue. Of the 33 articles selected for eligibility, 2 narrative reviews [23,24] and 2 conference papers [25,26] were excluded as they did not meet the predefined inclusion criteria in terms of study design, although thematically relevant to the topic. Overall, 29 experimental studies on mouse models were included in our systematic review, as shown in the flow chart in Fig 1.

### Risk of bias and level of certainty of the evidence

**Fig 2:**
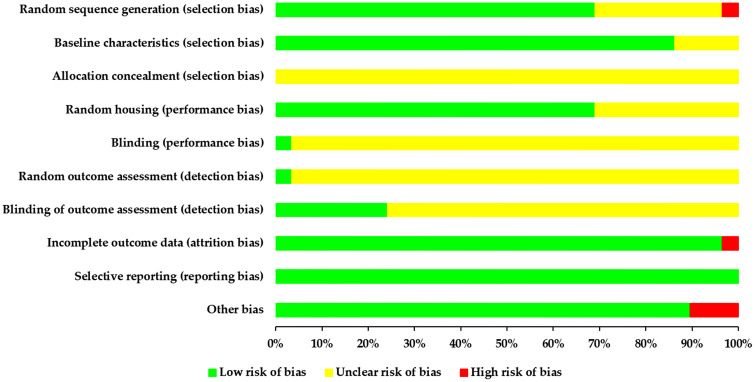
Assessment of methodological quality and risk of bias using the RoB tool for animal intervention studies (SYRCLE’s RoB) tool. Each of the ten domains was rated as “yes” for low risk (green), “no” for high risk (red), or “unclear” when information was missing (yellow).

SYRCLE’s RoB tool was used by three reviewers independently to assess the risk of bias and methodological quality of the included studies. Fig 2 shows that none of the included studies were judged to be at low risk of bias for all quality standards.

**Fig 3:**
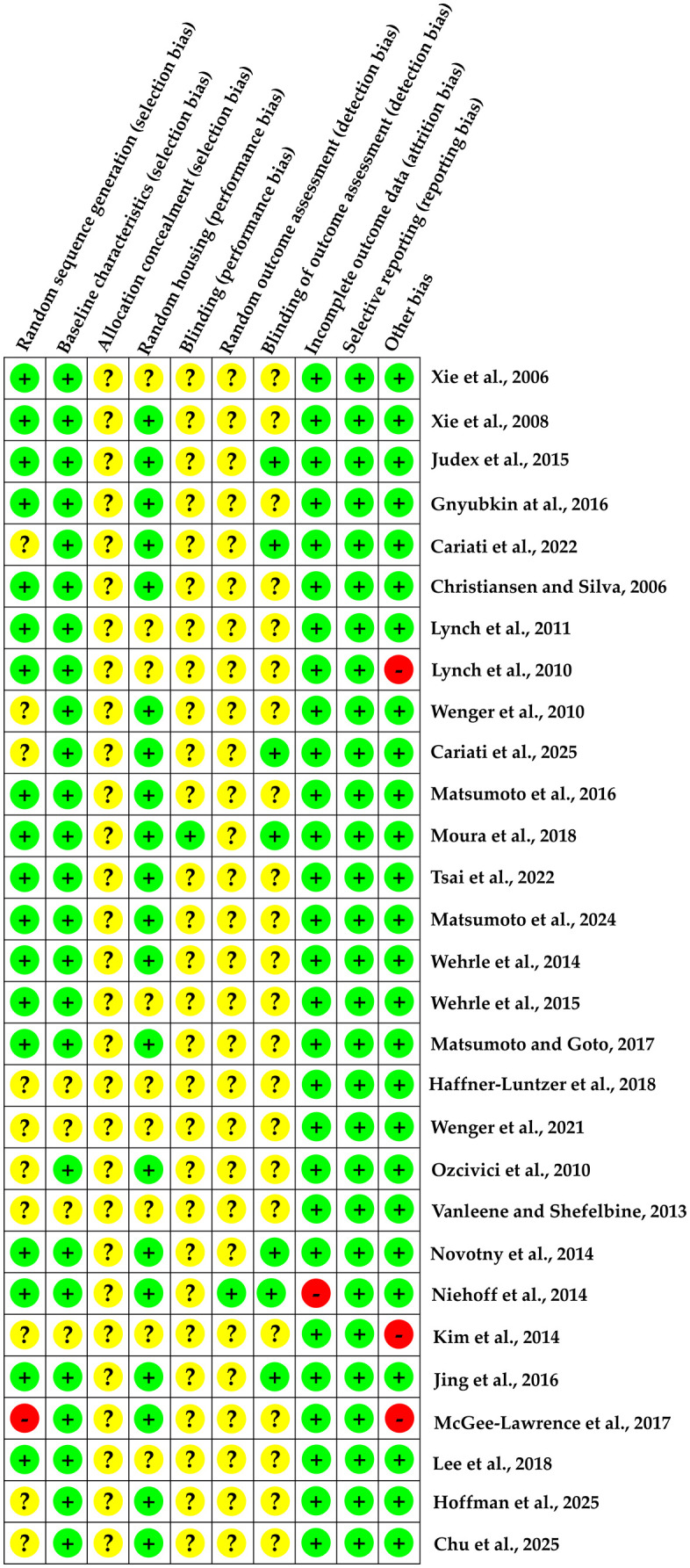
Individual assessment of methodological quality and risk of bias for the 29 included studies. Green indicates low risk, red high risk, and yellow unclear risk.

About selection bias, 69% (20/29) of the studies used adequate methods for random sequence generation; 28% (8/29) of the studies did not sufficiently describe the procedure, posing an unclear risk, while in only one study (3%, 1/29) were the experimental groups balanced by weight (random sequence generation). Almost all studies (86%, 25/29) show homogeneous groups at baseline; the remaining 14% (4/29) do not provide sufficient information, leading to an unclear risk (baseline characteristics). None of the studies (100%, 29/29) described adequate procedures for allocation concealment. In terms of performance bias, 69% (20/29) of studies reported random housing of animals during the experimental procedure, while 31% (9/29) did not provide sufficient information (random housing). For the fifth domain (blinding of caregivers/investigators), only 3% (1/29) of studies reported adequate blinding of researchers or staff involved, as in 97% (28/29) of cases, the information was insufficient, posing an unclear risk. Regarding detection bias, 97% (28/29) of the studies did not describe the method of random selection of animals in the evaluation of results (random outcome assessment), and only 3% (1/29) reported random assignment of outcomes. For the seventh domain (blinding of outcome assessment), 24% (7/29) of studies reported adequate blinding of the outcome assessment; in most cases (76%, 22/29), this information was insufficient, posing an unclear risk. About attrition bias, 97% (28/29) of the included studies provided complete outcome data, while only one study (3%, 1/29) deliberately omitted this information, creating a high risk of bias (incomplete outcome data). All studies (100%, 29/29) did not selectively report results, indicating a low risk of bias for this domain (selective reporting). Finally, 90% (26/29) of studies did not show other sources of bias, indicating a low risk. Only three studies (10%, 3/29) had potential additional sources of bias, indicating a high risk. The specific distribution of each risk of bias is shown in Fig 3.

Overall, the most critical domains include allocation concealment (selection bias), blinding (performance bias), random outcome assessment (detection bias) and blinding of outcome assessment (detection bias), mainly due to limited methodological detail. On the contrary, selective reporting (reporting bias) showed a low risk in all included studies, while the domains incomplete outcome data (attrition bias) and other bias predominantly showed a low risk. Random sequence generation and baseline characteristics (selection bias) also showed an adequate methodological profile in most studies. Therefore, the assessment of risk of bias using SYRCLE’s RoB tool highlights heterogeneous quality, with strengths in the domains related to data completeness and outcome selection, and limitations mainly in allocation and blinding procedures.

Regarding to the adapted GRADE approach, the certainty of the evidence was low for seven outcomes (trabecular bone volume, BV/TV; trabecular number, Tb.N; trabecular thickness, Tb.Th; cortical thickness, Ct.Th; bone formation rate, BFR/BS; mineralizing surface, MS/BS and cortical bone area, Ct.Ar) and very low for three outcomes (trabecular separation, Tb.S; bone mineral density, BMD and osteoclastic activity, Oc.S/BS). This was mainly due to the presence of indirectness in all the outcomes analyzed, while inconsistency and publication bias contributed to the reduction in certainty for some outcomes. Finally, the risk of bias was a downgrading factor for only two measures, contributing to the overall reduction in the certainty of the evidence.

### Summary of results

#### Effects of WBV on bone tissue in young, adult, and elderly mice.

WBV induces heterogeneous effects on bone tissue, with responses differing across young, adult, and aged healthy mice. The studies reporting these findings are summarized in Table 2 [27–36].

**Table 2:** A schematic representation of the WBV effects on bone tissue in different mouse models.

Reference	Mouse model	Age/sex/sample size	WBV protocol	Comparator	Outcomes
[27]	BALB/cByJ	8-week-old female (n = 38)	- WBV group (n = 10): 15 min/day, 5 days/week for 3 weeks; frequency: 45 Hz; acceleration: 0.3 g - WBV-R group (n = 10): 15 min/day, 5 days/week for 3 weeks with 10-s rest periods after each second of vibration; frequency: 45 Hz; acceleration: 0.3 g	- BC group (n = 8): baseline control - AC group (n = 10): placed on an inactive plate for 15 min/day	- Reduced MAR and BFR/BS in AC mice compared to BC mice - Increased BFR/BS of endocortical bone in the WBV group compared to AC mice - Reduced Oc.S/BS in the trabecular metaphysis and trabecular epiphysis in the WBV group compared to AC mice - Increased BV/TV in the tibial epiphysis and Tb.Th in both the epiphysis and metaphysis in AC mice compared to BC mice
[28]	BALB/cByJ	8-week-old female (n = 32)	- WBV group (n = 12): 15 min/day, 5 days/week for 6 weeks; frequency: 45 Hz; acceleration: 0.3 g; shift: ∼74 μm	- BC group (n = 8): baseline control - AC group (n = 12): placed on an inactive plate for 15 min/day	- Increased TV and BV in the proximal tibia of AC and WBV mice compared to BC mice - Increased Ct.Ar, Ec.En, and Ps.En in the WBV group - Reduced MS/BS in AC and WBV mice compared to the BC group, with significant differences between the AC and WBV groups
[29]	BALB/cByJ	6-week-old male (n = 130)	0.3 g groups: - Group 1 (n = 11): 15 min/bout, 1 bout/day - Group 2 (n = 10): 15 min/bout, 2 bouts/day separated by 6 h - Group 3 (n = 12): 15 min/bout, 4 bouts/day separated by 3 h - Group 4 (n = 12): 30 min/bout, 1 bout/day - Group 5 (n = 11): 30 min/bout, 2 bouts/day separated by 6 h - Group 6 (n = 12): 60 min/bout, 1 bout/day 0.6 g groups: - Group 7 (n = 11): 15 min/bout, 1 bout/day - Group 8 (n = 11): 15 min/bout, 2 bouts/day separated by 6 h - Group 9 (n = 11): 30 min/bout, 1 bout/day All regimes were applied 5 days/week with two consecutive rest days for 3 weeks; frequency: 45 Hz	- BC group (n = 5): baseline control − 0 g group (n = 12): exposed to an inactive vibrating plate either once a day for 15 min - AC group (n = 12): exposed to an inactive vibrating plate twice a day for 30 min	- Increased BFR/BS in Groups 4, 6, and 7 compared to AC mice - Increased Oc.S/BS in Group 7 - Increased BV/TV and Tb.N in Group 2 compared to AC mice - Increased BV/TV in Groups 5 and 7 - Increased BFR/BS on the metaphyseal endocortical surface in Groups 2, 4, 5, and 7
[30]	C57BL/6J	7-week-old male (n = 58)	- WBV 3w group (n = 16): 15 min/day, 5 days/week for 3 weeks; frequency: 90 Hz; acceleration: 2 g - WBV 9w group (n = 8): 15 min/day, 5 days/week for 9 weeks; frequency: 90 Hz; acceleration: 2 g	- BC group (n = 10): basal control - CTRL 3w group (n = 16): exposed to inactive shaker, 15 min/day, 5 days/week for 3 weeks - CTRL 9w group (n = 8): exposed to inactive shaker, 15 min/day, 5 days/week for 9 weeks	- Increased MAR, BFR/BS, and Oc.S/BS, both in the distal femoral metaphysis and in the L2 vertebral body, in the WBV 3w group compared to the CTRL 3w group - Increased Ct.Th and endosteal MAR in the WBV 3w group compared to the CTRL 3w group - Reduced number of sclerostin-positive osteocytes in the WBV 3w group compared to the CTRL 3w group
[31]	BALB/c	4-month-old male (n = 20)	- B-trained group (n = 5): 5 vibration series of 3 min each with 1 min of recovery, 3 days/week for 12 weeks; frequency: 45 Hz; acceleration: 2 g; shift: 1.5 mm - C-trained group (n = 5): 3 vibration series of 2 min and 30 s each with 2 min and 30 s of recovery, 3 days/week for 12 weeks; frequency: 45 Hz; acceleration: 2 g; shift: 1.5 mm	- CTRL SED group (n = 5): sedentary mice - CTRL WBV group (n = 5): subjected to motor sounds, but not exposed to vibratory training	- Increased BV/TV and Tb.Th in both trained groups, associated with a reduction in Tb.S, compared to sedentary animals - Increased expression of COL-1 in the bone tissue of trained animals, with more marked effects in the C-trained group
[32]	C57BL/6	7-month-old adult male (n = 40)	− 0.1 g (n = 10), 0.3 g (n = 10), 1.0 g (n = 10) groups: 15 min/day, 7 days/week for 5 weeks; frequency: 45 Hz; acceleration: 0.1 g, 0.3 g or 1.0 g	- CTRL group (n = 10)	- Increased BV/TV in the proximal tibial metaphysis and distal femoral metaphysis of 0.1 g and 1.0 groups compared to the CTRL group - Increased BV/TV in the L5 vertebra of 0.1 g and 1.0 g groups compared to the 0.3 g group - Increased Tb.Th in the proximal tibial metaphysis of 0.1 g and 1.0 g groups compared to the CTRL and 0.3 g groups - Increased Tb.Th in the L5 vertebra of 0.1 g and 1.0 g groups compared to the 0.3 g group
[33]	BALB/c	7-month-old male (n = 81)	− 0.3 g groups: 0 (n = 13), 10 (n = 14), or 40 (n = 13) μg/kg/day PTH intraperitoneally 30 min before WBV (15 min/day, 5 days/week for 8 weeks; frequency: 90 Hz; acceleration: 0.3 g)	− 0 g groups: 0 (n = 14), 10 (n = 13), or 40 (n = 14) μg/kg/day PTH intraperitoneally 30 min before sham loading	- Increased BMC and cortical bone volume in the mid-diaphysis after 8 weeks of PTH treatment, independent of WBV exposure - Reduced Tb.N and increased Tb.S in animals treated with PTH and exposed to WBV
[34]	BALB/c	7- and 22-month-old male (n = 75)	− 0.3 g (n = 12–13 for age) and 1.0 g (n = 12–13 for age) group: 15 min/day, 5 days/week for 5 weeks; frequency: 90 Hz; acceleration: 0.3 g or 1.0 g	- Sham group (n = 12–13 for age): exposed to sham loading	- Increased BMC of the lower leg only in the 0.3 g and 1.0 g groups at 7 months - No significant changes in BV/TV, Tb.Th, Tb.N, Tb.S, MS/BS and BFR/BS after WBV exposure - No significant changes in osteoclast surface, structure and cortical bone formation after WBV exposure
[35]	C57BL/6	18-month-old male (n = 36)	- LOW group (n = 9): 30 min/day, 5 days/week for 12 weeks; frequency: 32 Hz; acceleration: 0.5 g - HIGH group (n = 9): 30 min/day, 5 days/week for 12 weeks; frequency: 32 Hz; acceleration: 1.5 g	- TIME-0 group (n = 9): sacrificed at 18 months - CTRL group (n = 9)	- Slight increase in bone volume in the head and femoral neck of the LOW group compared to the CTRL and HIGH groups - Increased MS/BS in the LOW and HIGH groups compared to the CTRL group - Increased number of osteoclasts in the HIGH group compared to the other experimental groups
[36]	BALB/c	4-, 12- and 24-month-old male (n = 45)	- WBV groups (n = 5 for age): 3 vibration series of 2 min and 30 s each with 2 min and 30 s of recovery, 3 days/week for 12 weeks; frequency: 45 Hz; acceleration: 2 g; shift: 1.5 mm	- SED groups (n = 5 for age): sedentary mice - CTRL groups (n = 5 for age): subjected to motor sounds, but not exposed to vibratory training	- Improved bone microarchitecture after WBV exposure in all age groups - Increased BV/TV and Tb.Th in the WBV groups, associated with a reduction in Tb.S, compared to the control groups
[37]	C57BL/6J	9-week-old female (n = 48)	- w-OVX group (n = 10): 20 min/day for 18 days; frequency: 45 Hz; acceleration: 0.3 g - pw-OVX group (n = 10): 30 µg/kg/day PTH subcutaneously and exposed to WBV for 20 min/day for 18 days; frequency: 45 Hz; acceleration: 0.3 g	- Sham-OVX group (n = 8): underwent simulated bilaterally ovariectomized surgery - c-OVX group (n = 10): control - p-OVX group (n = 10): 30 µg/kg/day PTH subcutaneously	- Improved BV/TV, Tb.Th, Tb.N, Ct.Th, Ct.Ar and Ma.Ar in the p-OVX and w-OVX groups compared to the c-OVX group - Increased BV/TV and Conn.D in the pw-OVX group compared to c-OVX mice - Greater mineral maturity and greater hardness in the pw-OVX group compared to the c-OVX group
[38]	Swiss	3‐month‐old adult female (n = 50)	- MV group (n = 10): OVX + exposed to WBV for 30 min/day, 5 days/week for 60 days; frequency: 60 Hz; acceleration: 0.6 g; shift: 1.0 mm - MV + ET group (n = 10): OVX + 17β‐estradiol and exposed to WBV for 30 min/day, 5 days/week for 60 days; frequency: 60 Hz; acceleration: 0.6 g; shift: 1.0 mm	- Sham group (n = 10): underwent simulated bilateral ovariectomized surgery - CTRL group (n = 10): OVX + 10 µg/kg/day sunflower oil, 7 days/week for 60 days - ET group (n = 10): OVX + 10 µg/kg/day 17β‐estradiol, 7 days/week for 60 days	- Increased BMD in the ET and MV + ET groups compared to the CTRL group - Increased BV/TV in the ET and MV + ET groups, with higher values in the MV + ET group
[39]	C57BL/6J	12-week-old female (n = 31)	- OVX + HFrD + WBV group (n = 7): high-fructose diet and exposed to WBV for 30 min/session, 2 sessions/day, 4 hours apart, 5 days/week for 16 weeks; frequency: 13 Hz; acceleration: 0.68 g; shift: 2.0 mm	- Sham group (n = 8): underwent simulated bilateral ovariectomized surgery - OVX group (n = 8): underwent ovariectomized surgery bilaterally - OVX + HFrD group (n = 8): high-fructose diet for 16 weeks	- No significant variation in BMD, BV/TV, Tb.S, and Tb.N between the experimental groups
[40]	C57BL/6JJcl	10-week-old female (n = 36)	- cWBV group (n = 12): 7.5 min/day, 5 days/week; frequency: 45 Hz; acceleration: 0.3 g - rWBV group (n = 12): 3-s bouts, followed by 9-second rest intervals, repeated for 30 min/day, 5 days/week; frequency: 45 Hz; acceleration: 0.3 g	- Sham group (n = 12): sham-treated	- No significant difference in B.Vf, B.Th and B.Sep parameters between the different experimental groups on day 7 post-surgery - Increased B.Vf and reduced B.Sep on day 14 post-surgery only in the rWBV group
[41]	C57BL/6Crl	12-week-old female (n = 48)	− 35 Hz (n = 14) and 45 Hz (n = 14) groups: 20 min/day, 5 days/week; frequency: 35 Hz or 45 Hz; acceleration: 0.3 g	- CTRL group (n = 20)	- Increased BV/TV and Tb.N in the non-osteotomized femurs of the 35 Hz group - Reduced flexural rigidity, bone formation and BV/TV in the 45 Hz group compared to the CTRL and the 35 Hz groups
[42]	C57BL/6NCrl	41-week-old female (n = 97)	- Non-OVX LMHFV group (n = 21): sham-operated and exposed to vibratory training (20 min/day, 5 days/week; frequency: 45 Hz; acceleration: 0.3 g) - OVX LMHFV group (n = 20): underwent bilaterally ovariectomized surgery and exposed to vibratory training (20 min/day, 5 days/week; frequency: 45 Hz; acceleration: 0.3 g) - OVX + E2 LMHFV group (n = 8): underwent bilaterally ovariectomized surgery + subcutaneous 17β oestrogen pellets (0.025 mg per pellet) and exposed to vibratory training (20 min/day, 5 days/week; frequency: 45 Hz; acceleration: 0.3 g)	- Non-OVX non-LMHFV group (n = 19): sham-operated - OVX non-LMHFV group (n = 21): underwent bilaterally ovariectomized surgery - OVX + E2 non-LMHFV group (n = 8): underwent bilaterally ovariectomized surgery + subcutaneous 17β oestrogen pellets (0.025 mg per pellet)	- Reduced callus flexural stiffness and BV/TV in the non-OVX LMHFV group compared to the non-OVX non-LMHFV group - Reduced bone callus and increased fibrous tissue in non-OVX LMHFV mice sacrificed on day 21 post-surgery compared to the non-OVX non-LMHFV group - Increased BV/TV and improved callus mechanical performance in the OVX LMHFV group compared to the OVX non-LMHFV group - Increased BV/TV and callus flexural stiffness in the OVX + E2 non-LMHFV group compared to OVX mice not treated with estrogen - No improvement in fracture healing in the OVX + E2 LMHFV group compared to OVX + E2 non-LMHFV
[43]	C57BL/6	13-week-old male (n = 48)	- Vib group (n = 24): 20 min/day for 6, 9 or 12 days; frequency: 30 Hz; acceleration: 0.1 g	- Sham group (n = 24): sham-treated	- On day 9, increased B.Vf and improved bone regeneration with reduced vascularization in both experimental groups - On day 12, increased B.Vf and B.Th and reduced B.Sep in the Vib group compared to the Sham group
[44]	C57BL/6J WT, ERα-KO, ERβ-KO	12-week-old female (n = 86)	- Sham-OVX vibration group (WT n = 8, ERα-KO n = 8, ERβ-KO n = 6): sham-treated and exposed to WBV for 20 min/day for 5 days; frequency: 45 Hz; acceleration: 0.3 g - OVX vibration group (WT n = 8, ERα-KO n = 7, ERβ-KO n = 7): underwent bilaterally ovariectomized surgery and exposed to WBV for 20 min/day for 5 days; frequency: 45 Hz; acceleration: 0.3 g	- Sham-OVX sham-vibration group (WT n = 6, ERα-KO n = 6, ERβ-KO n = 8): sham-treated - OVX sham-vibration group (WT n = 7, ERα-KO n = 6, ERβ-KO n = 9): underwent bilaterally ovariectomized surgery	- Increased BV/TV, bony bridging, flexural rigidity of the fracture callus and ObS/BS in WT OVX vibration mice compared to the WT sham-OVX vibration group - No significant change in bone healing in ERα-KO mice - Increased BV/TV, bony bridging, flexural rigidity of the fracture callus, NOb/BPm and ObS/BS in ERβ-KO OVX vibration mice compared to the ERβ-KO sham-OVX vibration group
[45]	C57BL/6	12-week-old male (n = 48)	- Vibrated group (n = 24): 20 min/day for 21 or 42 days; frequency: 30 Hz; acceleration: 0.3 g	- CTRL group (n = 24)	- No significant variation in bone parameters and biomechanical properties between the various experimental groups
[46]	C57BL/6J (B6)	7-week-old male (n = 108)	Phase I - HU + VIB (n = 24): subjected to hindlimb unloading and exposed to WBV (15 min/day, 7 days/week for 3 weeks; frequency: 90 Hz; acceleration: 0.2 g) Phase II - RA + VIB (n = 12): subjected to free reambulation and exposed to WBV (15 min/day, 7 days/week for 3 weeks; frequency: 90 Hz; acceleration: 0.2 g) - VIB-HU group (n = 6): exposed to WBV (15 min/day, 7 days/week for 3 weeks; frequency: 90 Hz; acceleration: 0.2 g) only during hindlimb unloading but not during free reambulation - VIB-RA group (n = 6): exposed to WBV (15 min/day, 7 days/week for 3 weeks; frequency: 90 Hz; acceleration: 0.2 g) only during free reambulation but not during hindlimb unloading	Phase I (n = 24 for group): - AC group: age-matched control - HU group: subjected to hindlimb unloading for 3 weeks - HU+SHAM: subjected to hindlimb unloading and placed on an inactive plate for 15 min/day, 7 days/week for 3 weeks Phase II (n = 12 for group): - AC group: age-matched control - RA group: subjected to free reambulation for 3 weeks - RA+SHAM: subjected to free reambulation and placed on an inactive plate for 15 min/day, 7 days/week for 3 weeks	- Reduced BV/TV, MS/BS and BFR/BS in the proximal tibial metaphysis of the HU, HU+SHAM and HU + VIB groups compared to AC mice - Reduced Ob.S/BS in all HU groups - Increased Oc.S/BS in HU and HU+SHAM mice - Reduced MAR in HU and HU+SHAM groups - After 6 weeks, partial recovery of BV/TV in RA, RA+SHAM and RA + VIB groups, with greater effects in animals exposed to vibrations - No change in MS/BS, BFR/BS and Ob.S/BS between the AC and RA + VIB groups − 30% increase in trabecular bone volume and 9% increase in Ma.Ar in the VIB-HU group compared to the VIB-RA group
[47]	- Homozygous WT (B6C3Fe-a/a- + /+) - Homozygous oim (B6C3Fe-a/a-oim/oim)	3-week-old female (n = 48)	- Wild vib group (n = 12): 15 min/day, 5 days/week for 5 weeks; frequency: 45 Hz; acceleration: ± 0.3 g - Oim vib group (n = 12): 15 min/day, 5 days/week for 5 weeks; frequency: 45 Hz; acceleration: ± 0.3 g	- Wild sham group (n = 12): placed on an inactive plate 15 min/day, 5 days/week for 5 weeks - Oim sham group (n = 12): placed on an inactive plate 15 min/day, 5 days/week for 5 weeks	- Increased femoral CSA in the cortical bone of the Wild vib group - Increased femoral Ct.Th and CSA in the tibial diaphysis of the Oim vib group - Increased bone volume fraction in the proximal tibial trabecular bone in the Oim vib group compared to Oim sham mice - Improved bending stiffness in the Wild vib group compared to Wild sham mice
[48]	- WT C57Bl/10 - Mdx	3-week-old male (n = 48)	- WT vibrated group (n = 14): 15 min/day, 5 days/week for 8 weeks; frequency: 45 Hz; acceleration: 0.6 g - Mdx vibrated group (n = 11): 15 min/day, 5 days/week for 8 weeks; frequency: 45 Hz; acceleration: 0.6 g	- WT non-vibrated group (n = 12): placed on an inactive plate 15 min/day, 5 days/week for 8 weeks - Mdx non-vibrated group (n = 11): placed on an inactive plate 15 min/day, 5 days/week for 8 weeks	- No significant change in bone volume fraction, trabecular thickness, separation, or number between vibrated and non-vibrated mice, in both WT and Mdx groups
[49]	C57BL/6	12-week-old female (n = 60)	- BTX + WBV (n = 10): exposed to WBV (30 min/day, divided into 10 consecutive sets of 3 min each, 5 days/week for 4 weeks; frequency: 25 Hz; acceleration: 2.1 g; shift: 0.83 mm) beginning 1 day after BTX injection - BTX + WBV + IGF-1 (n = 10): exposed to WBV (30 min/day, divided into 10 consecutive sets of 3 min each, 5 days/week for 4 weeks; frequency: 25 Hz; acceleration: 2.1 g; shift: 0.83 mm) + 1 μg/day IGF-1 subcutaneously twice daily beginning 1 day after BTX injection for 4 weeks	- Baseline group (n = 10): sacrificed at the start of the study (day 0) - SHAM group (n = 10): 0.9% saline solution at day 0 - BTX group (n = 10): 1.0 U/0.1 mL BTX intramuscularly at day 0 - BTX + IGF-1 (n = 10): 1 μg/day IGF-1 subcutaneously twice daily beginning 1 day after BTX injection for 4 weeks	- Reduced Tb.BMD, Tb.BMC, Ct.BMD, and Ct.BMC in the distal femoral metaphysis of the BTX group compared to SHAM mice - Reduced Tb.BMD, Tb.BMC, Ct.BMD, Ct.BMC, Ct.CSA, and Ct.Th in the injected limb of the BTX group compared to the contralateral limb - Reduced Tb.BMD, Tb.BMC, Ct.BMD, Ct.BMC, Ct.CSA and Ct.Th in the injected limb of the BTX + WBV group compared to the contralateral limb, with significant values only for Ct.BMD compared to SHAM mice - No significant improvement induced by IGF-1 injection
[50]	BALB/c	6-week-old (n = 38)	- LV1 group (n = 6): 10 min/day for 3 days; frequency: 45 Hz; acceleration: 0.4 g, on the day after the first LPS injection - LV2 group (n = 6): 10 min/day for 3 days; frequency: 45 Hz; acceleration: 0.4 g, on the day after the second injection	- CTRL group (n = 13) - L group (n = 13): 5 mg/kg LPS by 2 intraperitoneal injections on days 0 and 4	- On day 7 after LPS injection, reduced bone volume and BMD in the tibia and in the femur in the L group compared to the CTRL group - On day 14 after LPS injection, reduced bone volume and BMD in the tibia and in the femur of the L group compared to the CTRL group - On day 7 after LPS injection, increased bone volume and BMD in the LV2 group compared to the L group
[51]	- db/db C57BKS - Homozygous WT	12-week-old male (n = 24)	- db/db + WBV group (n = 8): 1 h/day for 12 weeks; frequency: 45 Hz; acceleration: 0.5 g	- WT group (n = 8): control group - db/db group (n = 8): diabetic mice with fasting blood glucose higher than 16.7 mmol/L	- Reduced CTx-1 in the db/db + WBV group compared to WT mice - Improved BV/TV, BS/BV, Tb.N, Tb.S, Ct.Th, Ct.Ar, MS/BS, MAR, BFR/BS, maximum load, stiffness and hardness in the db/db + WBV group, with values like those of WT mice
[52]	db/db and WT C57Bl6/J	5-week-old male	- WBV groups (n = 14–16): 20 min/day for 12 weeks; frequency: 32 Hz; acceleration: 0.5 g	- SED groups (n = 14–16): sedentary animals - TE groups (n = 14–16): treadmill exercise with a 5% incline, 10 m/min, 45 min/day for 12 weeks	- Increased serum osteocalcin in db/db mice exposed to WBV or TE respect to SED db/db mice - Reduced BMD in the femoral neck in db/db mice compared to WT mice - No significant change in bone strength in trained db/db mice compared to the corresponding SED group
[53]	C57BL/6	5-week-old male (n = 24)	- NS + V group (n = 8): night shift and exposed to WBV (5 repetitions each lasting 35 seconds of vibration at 30 Hz, 20 sec at 40 Hz and 5 sec at 50 Hz, followed by 1 min of rest for a total of 30 min/day, 5 days/week for 4 weeks; acceleration: 0.3 g)	- Nor group (n = 8): control group - NS group (n = 8): night shift	- Reduced BMD, BV/TV, Tb.N and Conn.Dn in the NS group after 4 weeks - Increased Tb.Th, Tb.N and Conn.Dn in the NS + V group compared to the NS group
[54]	C57BL/6J	12-week-old male (n = 24)	- VML‐0.6 g (n = 8) and VML‐1.0 g (n = 8) groups: 3 days after the VML injury, 15 min/day, 5 days/week for 8 weeks; frequency: 45 Hz; acceleration: 0.6 g or 1.0	- VML-noTx group (n = 8): placed on an inactive plate 15 min/day, 5 days/week for 8 weeks	- Increased tibial cortical volume in the VML-0.6 g and VML-1.0 g groups compared to VML-noTx mice - Increased cortical diameter in the VML-0.6 g group compared to VML-noTx mice - Increased trabecular bone volume fraction, trabecular number, trabecular thickness and trabecular BMD in the tibiae of VML-0.6 g and VML-1.0 g mice compared to the VML-noTx group - Reduced trabecular spacing in the vibrated groups compared to VML-noTx mice
[55]	- BALB/c - C57BL/6J	− 8-week-old male (n = 20) − 31- to 36-week-old female (n = 25)	Young mice: - Veh Vibration group (n = 5): 5 mg/kg body weight of DMSO intraperitoneally 1 hour before WBV (two 15-min sessions, with a 15-min rest period, 5 days/week for 4 weeks; frequency: 13 Hz; acceleration: 0.3 g) - Yoda1 Vibration group (n = 5): 5 mg/kg body weight of Yoda1 intraperitoneally 1 hour before WBV (two 15-min sessions, with a 15-min rest period, 5 days/week for 4 weeks; frequency: 13 Hz; acceleration: 0.3 g) Mature mice: - Veh Vibration group (n = 3): 5 mg/kg body weight of DMSO intraperitoneally 1 hour before WBV (two 7.5-min sessions, with a 7.5-min rest period, 5 days/week for 4 weeks; frequency: 13 Hz; acceleration: 0.3 g) - Yoda1 Vibration group (n = 9): 5 mg/kg body weight of Yoda1 intraperitoneally 1 hour before WBV (two 7.5-min sessions, with a 7.5-min rest period, 5 days/week for 4 weeks; frequency: 13 Hz; acceleration: 0.3 g)	Young mice: Veh Ground group (n = 5): 5 mg/kg body weight of DMSO intraperitoneally - Yoda1 Ground group (n = 5): 5 mg/kg body weight of Yoda1 intraperitoneally Mature mice: - Veh Ground group (n = 5): 5 mg/kg body weight of DMSO intraperitoneally - Yoda1 Ground group (n = 8): 5 mg/kg body weight of Yoda1 intraperitoneally	In young mice: - No changes in BV/TV, Tb.N and Tb.S at the end of the 4-week experiment - Increased Ct.pMOI at the end of the 4 weeks only in the Yoda1 Ground group - Increased Ct.Th at week 4 only in the Veh Vibration group - Increased Ct.Formation Vol at week 2 in the Yoda1 Ground group and at week 4 in the groups subjected to vibration In mature mice: - Attenuated reduction in Ct.pMOI at week 2 in the group undergoing combination therapy compared to untreated controls - Improvemed Ct.Ar at week 2 in the Yoda1 Vibration group - Increased Ct.Formation.Vol at week 2 in the group undergoing combination treatment

WBV: whole-body vibration; MAR: mineral apposition rate; BFR/BS: bone formation rate; Oc.S/BS: osteoclastic activity; BV/TV: trabecular bone volume; Tb.Th: trabecular thickness; TV: bone marrow volume; BV: trabecular bone volume; Ct.Ar: cortical bone area; Ec.En: bone marrow area; Ps.En: periosteal area; MS/BS: mineralizing surface; Tb.N: trabecular number; Ct.Th: cortical thickness; Tb.S: trabecular separation; COL-1: type 1 collagen; BMC: bone mineral content; PTH: parathyroid hormone; OVX: ovariectomized; Tb.N: trabecular number; Ma.Ar: medullary area; Conn.D: trabecular bone connectivity density; BMD: bone mineral density; B.Vf: bone volume fraction; B.Th: bone thickness; B.Sep: bone spacing; LMHFV: low-magnitude high-frequency vibration; WT: wildtype; ERα-KO: homozygous B6.129P2-Esr1^tm1Ksk/J^ mice; ERβ-KO: homozygous B6.129P2-Esr2^tm1Unc/J^ mice; ObS/BS: osteoblast surface per bone surface; NOb/BPm: number of osteoblasts per bone perimeter; CSA: cross-sectional area; Mdx: mouse model of Duchenne muscular dystrophy; BTX: botulinum toxin A; IGF-1: insulin-like growth factor 1; Tb.BMD: trabecular bone mineral density; Tb.BMC: trabecular bone mineral content; Ct.BMD: cortical bone mineral density; Ct.BMC: cortical bone mineral content; Ct.CSA: cross-sectional area; LPS: lipopolysaccharide; TRACP5b: tartrate-resistant acid phosphatase 5b; BS/BV: trabecular bone surface/bone volume; CTx-1: C-terminal cross-linked telopeptides of type 1 collagen; TE: treadmill exercise; VML: mouse model of volumetric muscle loss; DMSO: dimethyl sulfoxide; Ct.pMOI: cortical polar moment inertia; Ct.Formation Vol: cortical bone formation volumes.

In young mouse models, vibratory training produces varied effects that are closely dependent on the characteristics of the protocol used, with a greater influence on bone remodelling processes than on bone formation alone. Particularly, some evidence has reported an association between WBV and reduced osteoclastic activity in trabecular bone, while changes in bone formation markers are less consistent and primarily limited to the cortical and endocortical levels [27,28]. In agreement, microstructural parameters show considerable heterogeneity across studies, with no clear association between the experimental model and the bone response to WBV [28–30]. Importantly, the osteogenic response appears to be strongly influenced by the duration of the mechanical stimulus, with increases in bone formation observed under conditions of prolonged exposure. On the other hand, short-term exposures can modulate the expression of bone turnover markers, without, however, leading to significant structural adaptations [29,30]. A key factor appears to be the recovery time between sessions, as it is associated with a greater osteogenic response and improvements in bone microarchitecture [31]. At the cellular and molecular levels, a reduction in sclerostin-positive osteocytes was observed, suggesting a modulation of the inhibitory pathways of bone formation. Furthermore, WBV appears to modulate the expression of type I collagen (COL-1), with effects on the quality of the newly formed bone matrix [30].

In adult mouse models, the response to WBV varies widely. Increases in trabecular bone mass and volume have been observed under specific stimulus intensity conditions, although most protocols do not reveal significant changes in microstructural parameters [32]. Furthermore, no synergistic effect was observed between WBV and anabolic drug treatments, such as the administration of parathyroid hormone (PTH), indicating that WBV has a limited ability to modulate bone remodelling in adulthood [33]. Accordingly, no significant changes were found in the microstructural parameters assessed by dual-energy X-ray absorptiometry (DXA), including bone volume, trabecular thickness and number, trabecular spacing, and bone mineral content (BMC), confirming that WBV has a minimal or undetectable effect in this age group [34].

Finally, overall modest effects of WBV were observed in aged mouse models. In some studies, slight improvements in microarchitectural parameters and markers of bone turnover were observed, while in others no significant differences were found compared to controls, suggesting reduced responsiveness of the skeleton to mechanical stimulation [35]. On the other hand, improvements in bone microarchitecture have been associated with specific experimental conditions, particularly when adequate recovery periods are allowed between sessions, suggesting that optimizing the protocol could partially preserve the osteogenic response even in aging individuals [36].

### Effects of WBV on bone tissue in mouse models of osteoporosis and on fracture healing

The effects of WBV on mouse models of osteoporosis and fracture healing are shown in Table 2 [37–45], with variable results depending on the pathological context, therapeutic combinations, and the characteristics of the protocol used.

In models of ovariectomy-induced osteoporosis, WBV appears to counteract bone mass loss and promote modest increases in structural and densitometric parameters. Specifically, a more consistent effect emerges from the combination of vibratory training with PTH or estrogen therapy, suggesting a possible synergy in improving the microarchitecture and biomechanical properties of bone tissue [37,38]. On the other hand, other studies have found no significant effects of WBV on structural and densitometric parameters even in the presence of nutritional interventions or prolonged exposure to vibrations, highlighting how the efficacy of the treatment may depend strictly on the characteristics of the experimental model and the mechanical stimulus applied [39]. Not surprisingly, protocols characterized by short recovery intervals between sessions appear to be more effective in promoting bone regeneration than continuous stimuli, confirming a key role for the temporal modulation of the mechanical vibration [40].

Importantly, the effects of WBV on fracture healing have been the subject of numerous studies. In various mouse models, exposure to WBV appears to improve bone regeneration and increase BMD at fracture sites, particularly in osteoporotic conditions or in the presence of hormonal changes [41,42,44]. In addition, low-intensity protocols may promote the early phase of bone repair by stimulating vascularization and the subsequent formation of callus [43]. However, these effects are highly dependent on the animal’s pathophysiological state and endocrine context, with different responses observed between ovariectomized (OVX) and non-OVX models [42]. Finally, some authors have not observed any significant improvement in fracture healing, either structurally or biomechanically, highlighting the lack of effect in specific stimulation protocols [45].

### Effects of WBV on other mouse models of bone loss

The effects of WBV on other models of bone loss are shown in Table 2 [46–55], with varied responses depending on the experimental conditions.

In disuse models, WBV is known to counteract bone loss and preserve trabecular microarchitecture compared to untreated controls, by reducing osteoclastic activity and promoting the maintenance of osteoblastic surface area. Furthermore, the osteogenic potential of the vibratory stimulus appears to be associated with the post-unloading recovery phase, accelerating the restoration of bone mass and formation [46].

In genetic models of bone fragility, such as osteogenesis imperfecta, exposure to mechanical vibrations promotes an increase in cortical thickness in long bones, with more pronounced effects in the diaphysis than in the metaphyseal regions. Moderate increases have also been observed at the trabecular level, in association with improved flexural stiffness, indicating an overall positive effect of WBV in this pathological condition [47].

In neuromuscular models, vibratory training does not induce significant changes in bone tissue, suggesting reduced bone responsiveness in the presence of severe musculoskeletal alterations [48,49]. Specifically, in the mouse model of Duchenne muscular dystrophy, no improvements were observed in terms of trabecular bone volume, trabecular number, and spacing in response to WBV, nor in the biomechanical properties already compromised by the disease [48]. Similarly, the vibratory stimulus only partially counteracts the skeletal damage characteristic of the model of bone loss induced by muscle paralysis via botulinum toxin A (BTX) injection, with slight improvements at the cortical level. Importantly, the association between WBV and anabolic factors, such as insulin growth factor 1 (IGF-1), did not further enhance the effects on bone tissue, suggesting a limited synergistic capacity of vibration in this model [49]. In agreement, a partial recovery of bone parameters was observed in the mouse model of lipopolysaccharide (LPS)-induced inflammatory, with improvements in bone mass and microarchitecture compared to controls, highlighting a key role of the intensity of the inflammatory process in responses to mechanical stimulation [50].

In metabolic models, such as those associated with leptin receptor deficiency, WBV improves trabecular and cortical microarchitecture, reduces the expression of bone resorption markers, and restores biomechanical properties [51]. In the same experimental model, a comparison with a group undergoing treadmill exercise shows a differential modulation of the bone response. Specifically, both vibratory training and exercise promote an increase in bone formation markers compared to sedentary controls, although BMD recovery is more pronounced in response to the active stimulus [52].

In models of circadian rhythm disruption, exposure to multi-frequency vibrations counteracted bone loss induced by a altered light-dark cycle, preserving trabecular microarchitecture and densitometric parameters compared to untreated animals [53]. Interestingly, positive effects of WBV were also observed in a murine model of volumetric muscle loss (VML)–induced musculoskeletal injury, including an increase in cortical bone volume and an improvement in trabecular microstructural parameters. These findings support the hypothesis that mechanical vibrations confer significant benefits to bone tissue following extensive muscle injury, suggesting its potential as a promising rehabilitative strategy for individuals with limited mobility [54].

Finally, in the mouse model of radiation-induced bone damage, the response to WBV appears to be limited and age-dependent. In this regard, young mice did not show significant changes in the structural parameters of trabecular bone following exposure to mechanical vibrations, while modest effects were observed primarily in cortical bone. On the other hand, transient changes that were not sustained over time were observed in adult animals. Furthermore, the association with pharmacological activation of the Piezo1 mechanosensitive channel activation via the agonist Yoda1 did not produce significant synergistic effects on bone tissue, highlighting the need for further studies on long-term effects and dose-dependent interactions between radiation, pharmacological stimulation, and mechanical vibrations [55].

## Discussion

Our systematic review aimed to summarize the effects of WBV on bone tissue in different mouse models, identifying the optimal conditions for maintaining bone mass and microarchitecture. Twenty-nine experimental studies were included, of which 10 were on healthy mice of different age groups, 9 on models of osteoporosis and fracture healing, and 10 on other models of bone loss. WBV showed generally favourable effects on bone formation, mineral density, and trabecular and cortical microarchitecture, although some evidence is conflicting, suggesting that efficacy depends on protocol parameters, age, skeletal site, and the underlying pathophysiological condition. This heterogeneity can be partly attributed to methodological differences across studies, both in terms of experimental protocols and completeness and detail of reporting, in line with what is frequently observed in preclinical research.

In young mice, WBV reduced resorption and improved mineralization. Xie et al. [27,28] observed significant improvements in BFR/BS, Ct.Ar, Ec.En and Ps.En parameters with short daily sessions at a frequency of 45 Hz, while Judex and colleagues [29] reported that longer exposures significantly increase bone formation compared to short sessions. Gnyubkin et al. [30] and Cariati et al. [31] confirmed increases in trabecular and cortical parameters, highlighting how recovery times between vibration series significantly influence the osteogenic response. In contrast, the effects of WBV in adult and elderly mice are more heterogeneous. Christiansen and Silva [32] reported significant increases in BV/TV and Tb.Th in the proximal tibial metaphysis and L5 vertebra, while other skeletal regions, such as the femoral condyles and proximal femur, showed no significant changes, suggesting a response dependent on both load intensity and anatomical site. Lynch et al. [33,34] and Wenger et al. [35] observed limited effects, as mechanical vibrations did not enhance the anabolic action of PTH or induce marked changes in bone mechanical properties. On the other hand, Cariati and colleagues [36] showed that adequate recovery times between vibration series can promote significant improvements in microarchitecture even in adult and elderly mice, confirming the importance of protocol optimization. Overall, bone adaptations to WBV in healthy models appear to be strongly age-dependent, with young mice showing greater sensitivity than adult and elderly mice. This may be due to the vibrational stimulus exerting a different influence on mechanotransduction processes and on the molecular pathways involved in modulating osteoblastic and osteoclastic activity. In this context, recent experimental evidence has suggested the involvement of fibronectin type III domain-containing protein 5 (FNDC5), NADPH oxidase 4 (NOX4), and sirtuin 1 (SIRT1) in the age-dependent differential response to WBV [36]. Furthermore, the modulation of COL-1 induced by WBV suggests a role for it in the quality and organisation of the newly formed bone matrix [31], confirming the existence of a complex interaction between mechanical and biochemical signals underlying the bone response to vibratory training.

In osteoporosis models, WBV combined with PTH or oestrogen showed significant synergistic effects on BV/TV, Conn.D and bone hardness [37,38]. In contrast, Tsai et al. [39] found that a low-frequency, long-duration protocol was not sufficient to restore bone parameters in OVX models fed a high-fructose diet, whereas WBV cycles with short rest intervals appeared to improve osteoporotic bone repair [40]. Wehrle and colleagues [41,42] observed a significant improvement in fracture healing in OVX mice, while WBV may be less effective or even detrimental in non-OVX mice, suggesting caution in clinical application. Subsequent studies on cortical perforation and iatrogenic fracture models [43–45] confirmed that WBV accelerates callus formation and improves mineral density, with effects modulated by age, fracture type, protocol, and hormonal status. Therefore, bone tissue with a high turnover rate or undergoing repair may show a greater responsiveness to vibratory stimulation. However, the considerable heterogeneity among the experimental models highlights a strong dependence on the pathophysiological context and bone remodelling pathways.

In models of bone loss due to disuse, WBV preserved bone formation and reduced osteoclastic activity. Ozcivici et al. [46] observed significantly higher MS/BS and BFR/BS values in mice subjected to limb unloading and WBV, suggesting an anabolic effect of the vibratory stimulus and faster recovery of bone mass during free walking. In genetic models of bone fragility, such as osteogenesis imperfecta, WBV showed more pronounced effects on cortical bone than on trabecular bone. Vanleene and Shefelbine [47] observed significant increases in Ct.Th and CSA in the femoral and tibial diaphysis of vibrated oim mice, while trabecular parameters of the distal femoral metaphysis were unchanged. In metabolic models, such as leptin receptor deficiency diabetes, WBV showed more comprehensive effects. Jing and colleagues [51] reported that WBV restored trabecular and cortical parameters and improved bone biomechanical characteristics in db/db mice. Similarly, McGee-Lawrence et al. [52] reported increased osteocalcin levels in db/db mice, although total femoral BMD was not completely restored. Effective protocols involved daily exposures at a frequency of 32–45 Hz and acceleration of 0.5 g, confirming that duration and intensity of the stimulus are critical parameters for the effectiveness of WBV. On the other hand, Novotny et al. [48] did not observe significant improvements in the bone structure of mdx mice, while Niehoff et al. [49] and Kim et al. [50] reported partial effects. Furthermore, Chu et al. [55] observed that the application of WBV and Yoda1, individually or in combination, in young and adult mice exposed to radiation, resulted in transient increases in cortical parameters such as Ct. Th and Ct.Formation Vol, without significant changes in the resorption-to-formation ratio or trabecular parameters, highlighting the need to investigate the long-term effects of the different treatments. In contrast, more recent models, such as those of circadian rhythm disturbance [53] or musculoskeletal injuries due to volumetric loss [54], have confirmed a protective effect of WBV, preserving microarchitecture and counteracting bone loss induced by physiological or traumatic stress. In fact, multi-frequency WBV protocols with daily sessions of 15–30 minutes have shown greater efficacy, suggesting that optimizing frequency and duration can maximise bone benefits.

The data available in humans support some of the effects of WBV observed in mouse models, highlighting the translational potential of preclinical findings. The meta-analysis by Elena Marín-Cascales et al. of 462 postmenopausal women under the age of 65 showed significant improvements in BMD of the lumbar spine and femoral neck after exposure to WBV compared to the control group [56]. Similarly, the meta-analysis by DadeMatthews and colleagues on 30 RCTs found an overall improvement in BMD in healthy postmenopausal women undergoing WBV protocols, both with vertical and side-alternating platforms, although no significant changes in bone formation and resorption biomarkers were observed [57]. More recently, Li et al. showed that a WBV protocol administered three times a week for 12 weeks, combined with vitamin D supplementation, resulted in significant increases in lumbar and femoral BMD in 48 elderly osteosarcopenic individuals, as well as improving appendicular muscle mass, strength and physical function. At the same time, increases in bone formation biomarkers and decreases in resorption biomarkers were observed, suggesting a synergistic effect of mechanical vibrations on the musculoskeletal system [58]. In contrast, the RCT by Maïmoun et al. on 14 subjects with spinal cord injury (SCI), who underwent a WBV protocol twice a week for 6 months (30–45 Hz, 0.5 g), did not show significant changes in total BMD or bone turnover markers, although a reduction in total fat mass was observed, suggesting reduced sensitivity of bone tissue to mechanical stimulation in particular chronic pathological conditions [59]. Similarly, the RCT by Högler and colleagues on 24 children with osteogenesis imperfecta exposed to WBV twice a day for 5 months showed no significant changes in BMD, muscle function, mobility or balance, although a significant increase in total lean mass was found compared to the control group. Therefore, in genetic conditions of bone fragility, WBV may have limited effects on skeletal tissue, highlighting the need for adapted protocols or combined approaches to achieve clinically relevant benefits [60]. Notably, studies in humans investigating the mechanistic and biological aspects of the response to WBV are even more limited and less consistent compared with preclinical models. Indeed, the available literature is relatively scarce, and only a few reports have directly explored WBV-induced changes in bone remodelling markers. Among these, a reduction in N-telopeptide X normalized to creatinine (NTx/Cr) has been observed in postmenopausal women exposed to 20 min of intermittent vibration for 1 or 3 weeks, whereas no changes in alkaline phosphatase (ALP) expression were detected [61]. In agreement, in a randomized crossover study in young men, resistance exercise combined with WBV induced acute changes in bone resorption markers, including C-terminal telopeptide of type I collagen (CTX-I) and tartrate-resistant acid phosphatase 5b (TRAP5b), while no changes were observed in bone formation markers [62]. Therefore, the biological mechanisms underlying the response to WBV in humans remain only partially characterized, highlighting a relevant translational gap with respect to preclinical animal models.

### Limitations of the study

Although the systematic review was conducted in accordance with the PRISMA guidelines and across three electronic databases, the study has certain limitations. Firstly, the heterogeneity of the WBV protocols, experimental parameters and measured outcomes limited the direct comparison of results, preventing a quantitative meta-analysis. In this regard, the experimental models used in the 29 included studies were organized into three sections to facilitate a structured synthesis of the evidence. However, marked variability was also observed within each group, particularly in terms of vibration frequency, acceleration, exposure duration, and characteristics of the vibrating platform. Such heterogeneity was also strongly reflected in the reported outcomes, which included microstructural, densitometric and biomechanical measures, as well as the expression of various bone turnover biomarkers, thereby preventing the execution of a quantitative synthesis even at the subgroup level. Furthermore, some studies reported incomplete details regarding protocols or measured parameters, introducing a potential risk of bias, as identified using the SYRCLE’s RoB tool. This methodological limitation was considered when interpreting the results and assessing the certainty of the evidence using an adapted GRADE approach, which indicated low or very low overall certainty for most of the outcomes. Finally, preclinical mouse models, whilst fundamental for investigating osteogenic mechanisms, do not fully replicate the complexity of human clinical conditions, limiting the generalizability of the results to clinical contexts.

### Strengths of the study

To our knowledge, this is the first systematic review to compile and compare evidence on the effects of WBV on bone tissue in different mouse models. The review included 29 studies on healthy, osteoporotic, disuse, genetic and metabolic mouse models, providing a comprehensive overview of the various experimental settings. Furthermore, the WBV protocols were reported in detail, in terms of frequencies, accelerations, session durations and recovery times, outlining the main trends in the results. This structured description allows for a comparative analysis of the experimental approaches used in the various studies, facilitating the identification of the main sources of methodological variability. Importantly, some preclinical data are supported by evidence in human populations, suggesting the translational relevance of the observations. However, the interpretation of this relevance requires extreme caution, given the significant differences between animal experimental models and human clinical conditions, particularly in terms of skeletal and biomechanical aspects. Overall, this systematic review provides a structured framework that may guide future studies aimed at optimizing WBV protocols and clarifying their possible clinical applications.

## Conclusions

WBV emerges as a potentially useful intervention for modulating bone turnover and improving density and microarchitecture in mouse models, with positive effects observed in most of the included studies. To our knowledge, this is the first systematic review that collects and compares evidence on the effects of WBV on bone in different mouse models. Despite the heterogeneity of experimental protocols and the lack of standardization of devices, most studies used frequencies between 32 and 45 Hz and accelerations between 0.1 and 0.6 g, while only a few reports provide shift measurements. However, the considerable variability in frequency, acceleration, shift, and exposure time across studies still prevents a clear understanding of the actual magnitude of the mechanical stimulus delivered, thereby limiting the robustness of the conclusions. This methodological variability highlights the need to define rigorous experimental protocols and instrumental descriptions to optimize the effectiveness of mechanical vibrations. Furthermore, optimizing frequency, acceleration and duration would appear to be crucial for maximizing the osteogenic response, although these findings remain preliminary and must be interpreted in the context of the limitations associated with animal models. Future studies should investigate the translational potential of WBV in clinically relevant contexts such as osteoporosis, age-related bone loss, rehabilitation and fracture healing, consolidating its role as an effective non-pharmacological strategy for maintaining bone mass and promoting skeletal health. Furthermore, research should improve the standardization and reporting of other key experimental parameters, such as waveform, loading direction and animal handling conditions, to enhance reproducibility and translational value. Finally, further studies should focus on the biological mechanisms underlying WBV-induced adaptations, which are currently poorly characterized, to improve understanding of the physiological processes involved and support a more robust interpretation of preclinical results.

## Supporting information

S1 Table Preferred Reporting Items for Systematic Reviews and Meta-Analyses (PRISMA) reporting checklist. (DOCX)

S2 Table Preferred Reporting Items for Systematic Reviews and Meta-Analyses (PRISMA) methodology. (DOCX)

S3 Table Certainty of evidence assessment using an adapted Grading of Recommendations, Assessment, Development, and Evaluation (GRADE) approach. (DOCX)
