# Supplementary material for: Bone physiological adaptations to whole-body vibration in mouse models: A Systematic Review
Source: PLoS One. 2026 Jul 14;21(7):e0353776. doi: 10.1371/journal.pone.0353776 (PMC13367732; doi:10.1371/journal.pone.0353776)
Supplement: S2 Table — (DOCX) [file pone.0353776.s002.docx]

**Table S1. Preferred Reporting Items for Systematic Reviews and Meta-Analyses (PRISMA) methodology.**

| **SEARCH STRATEGY** | | ((Whole Body Vibration) OR (Whole-Body Vibration) OR (WBV) OR (Vibratory Training)) AND ((Musculoskeletal system) OR (Bone) OR (Bone Mineral Density) OR (BMD) OR (Bone Loss) OR (Bone Mineral Content)) AND ((Mouse Models) OR (Murine Models) OR (Mice)) | |
| --- | --- | --- | --- |
| **SEARCH DATE** | | 2 January 2026 | |
| **FILTERS APPLIED** | | None | |
|  | | | |
| **STAGE** | | **RECORDS (n)** | **NOTES** |
| **Identification** | Records identified | 221 | MEDLINE (n=107)  Scopus (n=66)  Web of Science (n=48) |
|  | After duplicates removed | 141 | 80 duplicates removed via Rayyan software |
| **Screening** | Excluded at screening for title and abstract | 108 | Non relevant to WBV or mouse bone models |
|  | Full-text assessed | 33 | Eligibility assessment |
|  | Full-text excluded | 4 | 2 narrative reviews  2 conference papers |
| **Included** | Included studies | 29 | Final included experimental studies |
