# Supplementary material for: Bone physiological adaptations to whole-body vibration in mouse models: A Systematic Review
Source: PLoS One. 2026 Jul 14;21(7):e0353776. doi: 10.1371/journal.pone.0353776 (PMC13367732; doi:10.1371/journal.pone.0353776)
Supplement: S3 Table — (DOCX) [file pone.0353776.s003.docx]

**Table S2. Certainty of evidence assessment using an adapted Grading of Recommendations, Assessment, Development, and Evaluation (GRADE) approach.**

| **Number of studies** | **Risk of bias** | **Inconsistency** | **Indirectness** | **Imprecision** | **Publication bias** | **Certainty** |  |
| --- | --- | --- | --- | --- | --- | --- | --- |
| **BV/TV** | | | | | | | |
| 17 | Not serious | Not serious | Serious | Not serious | Not serious | **Low** |  |
| **Tb.N** | | | | | | | |
| 9 | Not serious | Serious | Serious | Not serious | Not serious | **Low** |  |
| **Tb.S** | | | | | | | |
| 7 | Not serious | Very serious | Serious | Not serious | Serious | **Very low** |  |
| **Tb.Th** | | | | | | |  |
| 7 | Not serious | Not serious | Serious | Not serious | Not serious | **Low** |  |
| **Ct.Th** | | | | | | |  |
| 6 | Not serious | Not serious | Serious | Not serious | Serious | **Low** |  |
| **BFR/BS** | | | | | | |  |
| 6 | Not serious | Not serious | Serious | Not serious | Serious | **Low** |  |
| **BMD** | | | | | | |  |
| 6 | Serious | Very serious | Serious | Not serious | Serious | **Very low** |  |
| **MS/BS** | | | | | | |  |
| 5 | Serious | Serious | Serious | Not serious | Not serious | **Low** |  |
| **OcS/BS** | | | | | | |  |
| 4 | Not serious | Very serious | Serious | Not serious | Not serious | **Very low** |  |
| **Ct.Ar** | | | | | | |  |
| 4 | Not serious | Not serious | Serious | Not serious | Not serious | **Low** |  |

BV/TV: trabecular bone volume; Tb.N: trabecular number; Tb.S: trabecular separation; Tb.Th: trabecular thickness; Ct.Th: cortical thickness; BFR/BS: bone formation rate; BMD: bone mineral density; MS/BS: mineralizing surface; Oc.S/BS: osteoclastic activity; Ct.Ar: cortical bone area.
